# Built environment and active play among Washington DC metropolitan children: A protocol for a cross-sectional study

**DOI:** 10.1186/s13690-015-0070-3

**Published:** 2015-04-27

**Authors:** Jennifer D Roberts, Rashawn Ray, Amber D Biles, Brandon Knight, Brian E Saelens

**Affiliations:** Department of Preventive Medicine and Biometrics, Division of Occupational and Environmental Health Sciences, F. Edward Hebert School of Medicine, Uniformed Services University of the Health Sciences, Bethesda, MD USA; Department of Sociology, University of Maryland, College Park, Maryland, USA; U.S. Naval War College, Newport, RI USA; Department of Pediatrics and Psychiatry & Behavioral Sciences, University of Washington and Seattle Children’s Research Institute, Seattle, WA USA

**Keywords:** Playability, BEAP Study, Built environment, Active play, Physical activity

## Abstract

**Background:**

Research has demonstrated that children who participate in active play are more likely to be physically active, thereby improving long-term health outcomes. Many adult studies have also shown that neighborhood built environments can encourage or discourage routine physical activity. Limited evidence has demonstrated that children who reside in neighborhoods with a built environment that is more inviting to active play exhibit lower overweight and obesity rates as well as an overall better state of well-being. This Built Environment and Active Play (BEAP) Study aims to develop a neighborhood playability rating system in the Washington, DC (DMV) area. Similar to walkability scores, these playability scores will estimate how affable a neighborhood is to active play. The BEAP Study will attempt to provide a broad view of factors influencing the level and type of active play among children.

**Methods/Design:**

Using a cross-sectional design, the BEAP Study will collect data using a mail questionnaire administered to the parents and/or guardians of 2000 children aged 7-12 years residing in select DMV areas in October of 2014. Questionnaire data, including information on active play, home and neighborhood characteristics, parental perceptions, and sociodemographic characteristics will be merged through a geographic information system (GIS) with objective built environment measures in the participants’ neighborhoods. An ordered logit model will be used to regress an ordinal active play outcome on built environment exposure variables while adjusting for potential confounders. Upon the construction of the final model, predictor coefficients will be used as parameters in the scoring system to develop neighborhood playability scores.

**Discussion:**

The BEAP Study intends to generate a neighborhood playability index by characterizing and quantifying children’s active play using parent-reported physical activity data in children, GIS data and built environment measures in participant neighborhoods. The BEAP Study will improve our understanding of the built environment and childhood playability relationship while also contributing to the body of evidence-based built environment and physical activity research.

**Electronic supplementary material:**

The online version of this article (doi:10.1186/s13690-015-0070-3) contains supplementary material, which is available to authorized users.

## Background

In the past 30 years, the prevalence of childhood obesity has more than doubled in children and quadrupled in adolescents [1,2]. In Washington, DC and the surrounding areas of Maryland, and Virginia (DMV), the prevalence rates of childhood obesity or overweight are 35% (Washington, DC), 31.6% (Maryland) and 29.8% (Virginia) [3]. Although poor nutrition is contributing to these rates of childhood overweight and obesity, the lack of physical activity also plays a role. Physical inactivity is increasing at disparate levels especially among some race and ethnic populations, adolescent girls, and lower socioeconomic groups [4,5]. The “2008 Physical Activity Guidelines for Americans” recommends that children have 60 minutes or more of physical activity each day, however, more than 80% of children and adolescents do not engage in enough aerobic physical activity to meet these guidelines [6-9]. In 2013, it was reported that only 16.4%, 21.6%, and 23.8% of the children and adolescents in Washington, DC, Maryland, and Virginia, respectively, were physically active for at least 60 minutes per day on the prior seven days and, nationwide, only one in three children report that they are physically active every day [8,10].

Research has demonstrated that children who participate in moderate-to-vigorous physical activity (MVPA) have improved long-term physical as well as mental health outcomes [11]. Studies in adults have shown that living in neighborhoods characterized by a poor quality built environment (e.g. limited green spaces and recreational facilities) is associated with a greater likelihood of depression and lower levels of physical activity [12-17]. Within this same age group, it was also found that as the amount of neighborhood park space increased, the level of physical activity increased [18]. Despite a growing body of research-based evidence, the relationship between the built environment and childhood recreational physical activity is still unfolding. The limited evidence has demonstrated that children who reside in neighborhoods with a higher quality built environment, or one that is more inviting to play engagement exhibit lower overweight and obesity rates as well as an overall better state of well-being [16,17]. Yet, recent built environment and physical activity research in children and adolescents have also shown some findings that are antithetical to what had been revealed in adults. For example, it has been observed that for adolescent children, as street connectivity of a neighborhood increased or as the density of cul-de-sacs decreased, the level of physical activity also decreased [18-20]. In contrast, for adults, higher levels of neighborhood walkability and lower cul-de-sac density have been identified with higher physical activity rates [21-25]. One of the underlying reasons for these contradictory findings between adults and children is that active transport is a significant component of adult physical activity while a much greater proportion of children’s physical activity consist of active play [26,27]. The same built environment characteristics that encourage adults’ active transport may inhibit childhood active play because of traffic or safety concerns. For the purpose of this Built Environment and Active Play (BEAP) Study, physical activity will be defined as active play, which is further being defined as participating in vigorous-intensity or moderate-intensity activities for fun and enjoyment in an official (e.g. team sports) or unofficial capacity (e.g. neighborhood game of basketball). This distinction is refined for children because active play, such as jumping rope or climbing trees, is their primary mode of physical activity and it is assumed that there is an underlying level of fun and enjoyment [28], while older populations of adolescents and adults are often more motivated to engage in physical activity for health or weight loss objectives [29,30]. Furthermore, it has been found that as children age and progress through adolescence, physical activity decreases by 60-70% (e.g. 9 year old girls median physical activity - 43.7 minutes per day vs. 15 year old girls median physical activity - 14.9 minutes per day) and sedentary behavior exceeds over 420 minutes or seven hours a day [31-33].

There has been research examining the reduction of physical activity in the school setting, but there is a paucity of literature on the association of outdoor physical activity or neighborhood active play with the built environment and other social determinants. Some studies have examined this issue in the Seattle, Washington, and San Diego, California areas, however, to date there has not been any study performed in the DMV [16,17,34]. The DMV has a residential population of over 5.8 million and is unique in that it is exceptionally heterogeneous with respect to race/ethnicity, income, birth origin and a variety of built environment variables, which may be contributing to the disparity rates of childhood physical activity, overweight and obesity. The BEAP Study will attempt to provide a broad view of factors, specifically neighborhood and/or social factors, influencing the level and type of active play among children. This will be achieved by developing a neighborhood playability rating system. Similar to walkability scores, these playability scores will estimate how conducive a neighborhood is to active play. Data obtained from this research study can provide vital information to community planners and to parents as they make residential location choices.

## Methods/Design

The cross-sectional study checklist for the Strengthening the Reporting of Observational Studies in Epidemiology (STROBE) was used as a guideline to structure and guarantee the presence of all recommended elements within this section.

### Study design

**Figure 1:**
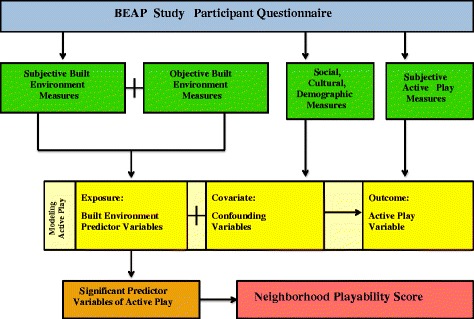
**BEAP study research design.**

Using an observational, cross-sectional design, this study will collect data through mail delivery of the BEAP Study questionnaire, which will be administered to the parents and/or guardians of 2000 children between the ages of 7-12 years residing in select areas of the DMV. The development of the BEAP Study questionnaire used in this study was adapted from a research instrument created in 1999 for the Neighborhood Impact on Kids Project that underwent several iterations of reliability and validity testing, including cognitive interviewing, and has been used in similar research studies [35,36]. An additional file provides the BEAP Study questionnaire in its entirety (See Additional file 1). Data from the BEAP Study questionnaire including information on active play, physical activity behaviors, home and neighborhood characteristics, parental neighborhood perceptions, and sociodemographic characteristics will be merged with geospatial data and objective measures of the built environment in the participants’ neighborhoods using their residential mailing address to model neighborhood playability scores (Figure 1). Selection of participants will occur in the DMV area using addresses purchased from Alesco Data Group, a direct marketing services company that provides mailing lists, data and related services for direct mail research. Upon receipt of the BEAP Study questionnaire, each participant will receive a $10 gift card redeemable at a pharmacy/general merchandise retail establishment, as well as, a postage-paid self-addressed envelope for the return of the completed BEAP Study questionnaire with a specified return date. Participants will also have the option of completing an identical online version of the BEAP Study questionnaire via Qualtrics.com with a provided secure and encrypted web address and unique access code.

### Setting

**Figure 2:**
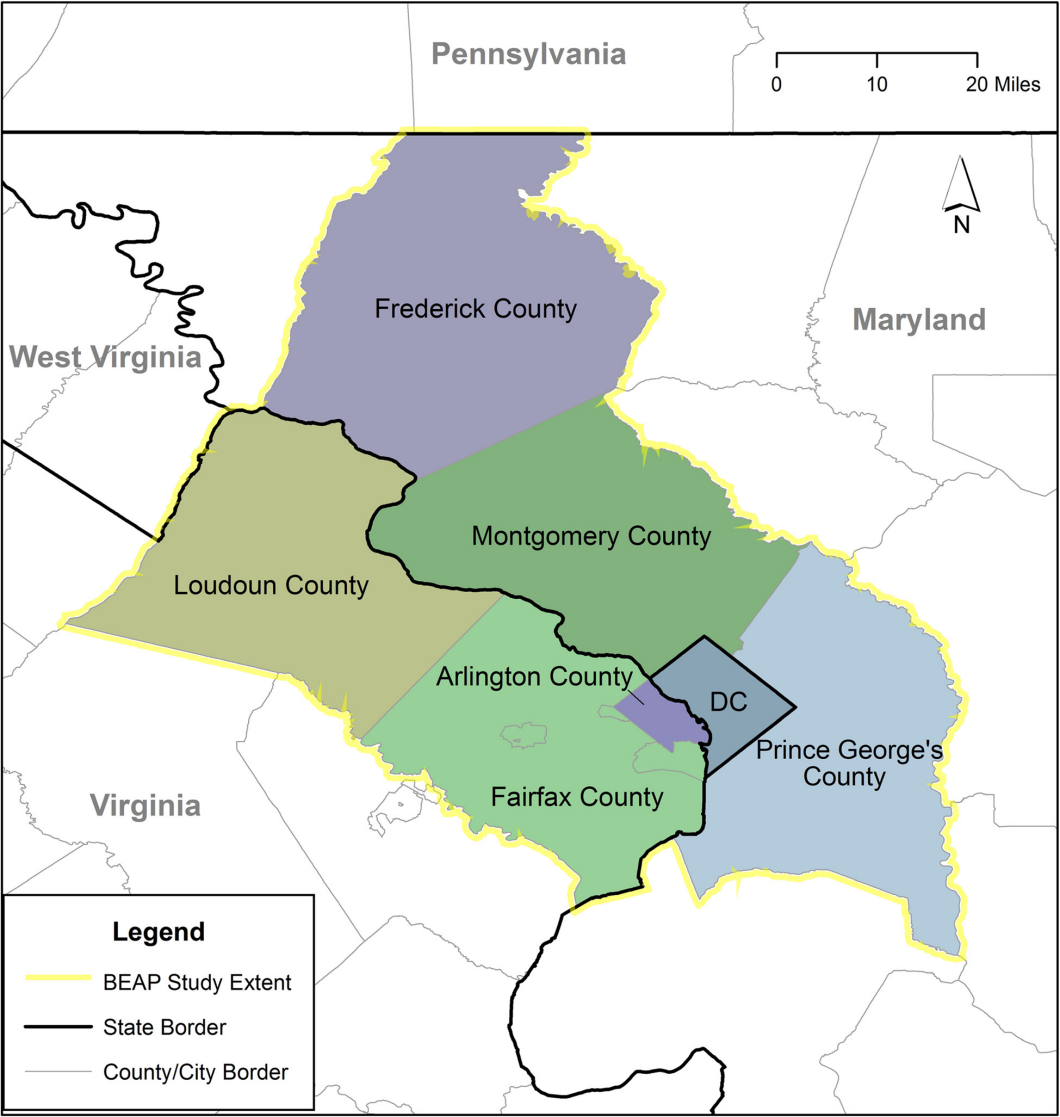
**BEAP study area map.**

Washington, DC (District of Columbia) and six contiguous counties were selected from the DMV area for inclusion in the BEAP Study (Figure 2). Within these major DMV areas, authoritative data containing parks and open space location are readily obtainable from local government sources, which will provide the foundation for geographic information system (GIS) analysis. Data will be collected from eligible households in October of 2014 within the geographic borders of (1) Washington, DC; (2) Fairfax County, VA; (3) Arlington County, VA; (4) Loudon County, VA; (5) Montgomery County, MD; (6) Prince George’s County, MD; and (7) Frederick County, MD (Table 1). The BEAP Study area mirrors the larger DMV with respect to sociodemographic characteristics and built environment features including residential density, green and open spaces, street intersection density, and urban form of neighborhoods, all of which are expected to be significantly associated with active play.

**Table 1:** **DMV study setting areas**

**Location**	**Population size**	**Population density (Persons per square mile)**
Washington, DC	646,450	9,856
Fairfax County, VA	1,131,000	2,7667
Arlington County, VA	225,000	7,994
Loudon County, VA	350,000	606
Montgomery County, MD	1,017,000	1,978
Prince George’s County, MD	890,100	1,789
Frederick County, MD	241,400	354

### Built environment areas and participant selection

Street Smart Walk Score®, developed in conjunction with Walk Score®, will be used as a tool for the BEAP Study’s stratified sampling strategy [37]. Calculated using walking routes to nearby amenities and road connectivity metrics, such as block length and intersection density, Street Smart Walk Scores® will be obtained for randomly selected street location(s) using latitude and longitude coordinates within the U.S. Census block groups of the seven major DMV areas. This BEAP Study area consists of 2901 block groups with varying population densities and land area sizes. The number of Street Smart Walk Scores® assigned to each block group will range from 1 (high population density-small land area) to 16 (low population density-large land area) depending on the total street length of the block group. Finally, the median Street Smart Walk Score® of the randomly selected location(s) will be used to assign each block group into one of five built environment classes using the classification scheme developed by Walk Score®: (1) Walker’s Paradise (90-100 score); (2) Very Walkable (70-89 score); (3) Somewhat Walkable (50-69); (4) Somewhat Car-Dependent (25-49); and (5) Very Car-Dependent (0-24) [37]. Potential participants from the BEAP Study area will be stratified into one of five strata based on the block group of their residential location. The use of the Street Smart Walk Score® for stratified sampling will help ensure adequate coverage of exposure to varying built environments [38].

Potential participants will be involved in one voluntary data collection activity, which will entail questionnaire data. Pre-eligibility will be assessed though the purchase of 2000 household addresses in the DMV of parents and/or guardian of children aged 7-12 years. Alesco Data Group has approximately 1.9 million DMV addresses and 150,000 unique addresses that meet this study’s age criteria from the study area. Addresses will be purchased from the five strata proportional to the population of households with children as estimated by the U.S. Census Bureau [39]. Prior to the mailing of the BEAP Study questionnaire, a brief pre-notice letter will be sent to the potential participants three days prior to the BEAP Study questionnaire mailing, which will describe the study and provide the study staff contact information in the event respondents have questions prior to study participation. Reminder and/or thank you post cards will be mailed seven days after the BEAP Study questionnaires have been sent.

For this study, the face page of the mailed BEAP Study questionnaire will contain the information generally found in a consent form. There will be a statement on that face page that will indicate that by answering the questions and returning the BEAP Study questionnaire in the postage-paid self-addressed envelope, the participant is providing and documenting his/her consent. With the online BEAP Study questionnaire version, the same consent information will be provided.

### Sample size

To construct the playability index, the BEAP Study will use a multiple regression fixed model to determine the relative weighting for each of the built environment components. It has been estimated that a total sample of 520 participants recruited from the entire BEAP Study area would allow the detection of a large effect with 80% power. The calculated sample size assumes a two-tailed probability level of 5% and a regression model with 15 covariates explaining 20% of the outcome variance size (R^2^ = 0.2). Assuming a highly conservative 30% response rate, 2000 BEAP Study questionnaires will be sent to DMV residents to achieve an exceedance of this sample size (2000 x 0.30 = 600) [40]. In addition to the benefit mentioned previously, the BEAP Study’s stratified sampling scheme is expected to reduce sampling error and increase the power to detect associations between built environment factors and active play.

### Exposure study variables

#### Objective built environment measures

GIS data will facilitate the creation of objective built environment measures in the participants’ neighborhoods. These data will be used to characterize the built environment surrounding the home residence of each participant with varying buffer distances (e.g. ½ mile, 1 mile) that will be able to appraise the characteristics and heterogeneity between many exposure limits. For example, open space buffer zones can be created and applied to each home residence to determine the density, distance and location of parks relative to each participant’s home. A geographic database of potential childhood play locations and road networks across the study area will be created using county/city authoritative data sources as well as data obtained from the U.S. Census Bureau, U.S. National Park Service, and OpenStreetMap.org. Subsequently, three types of objective built environment measures will contribute to assessing the feasibility of active play, including (1) open recreational space; (2) street connectivity (block size; block length; and intersection density); and (3) net residential density (Table 2). Open recreational space will be defined as any land use area officially designated as a park or recreational area. Schoolyards and playgrounds will also be incorporated in this definition of open recreational space. Highly connected street networks create shorter and more abundant routes to destinations. Furthermore, increased connectivity has been associated with increased walkability while those that include longer blocks, fewer intersections, and more dead-ends may inhibit walking [41-45]. The relationship between street connectivity, including block size and block length, and playability may not be the same as the relationship with walkability, however street connectivity may still be a significant predictor of playability. Another measure of street connectivity is intersection density, which will be defined in this study as the number of intersections within a specified buffer zone. Intersection density is inversely related to block size and smaller blocks have been shown to make a neighborhood more walkable [41]. A recent study found that of all the built environment measurements, intersection density has the largest effect on walking, which may also have an impact on playability [46]. Residential density refers to the total residential units divided by the total residential land area. High (e.g. 10,000 ppsm) to medium (e.g. 1,000 ppsm) residential densities have been shown to be walk promoting environments when the purpose of walking is travel or leisure, but not overall physical activity [47].

**Table 2:** **Summary of study variables**

**Exposure study variables**	**Study covariates (continued)**
***Objective built environment Measures***	***Body size and health conditions***
Open recreational space	Child BMI
Block size	Parent BMI
Block length	Child anxiety
Intersection density	Child asthma
Residential density	Child ADHD/ADD
***Home and neighborhood characteristics***	***Child electronic availability and use***
Home building type	Presence of electronics in bedroom
Home yard	Ownership of electronics
Street sidewalks	Minutes/day of sedentary behavior
Street type	***Child active commuting***
***Neighborhood accessibility***	Frequency of active commuting
Education destinations	Duration of active commuting
Transport destinations	***Dog ownership***
Recreation destinations	Frequency of child dog walking
Food retail destinations	Frequency of child dog playing
Social destinations	***Parent rules***
**Outcome study variable**	Child’s homework rules
***Child active Play***	Child’s electronic use rules
Minutes/day of active play	Child’s outdoor play rules
Occurrence of active play	***Parent neighborhood perceptions***
**Study covariates**	Perceived neighborhood safety
***Demographics***	Perceived neighborhood quality
Child age	***Parent physical activity***
Child sex	Employment physical activity
Child race/ethnicity	Recreational physical activity
Child nativity	***Active play qualifiers***
Child education	Location of active play
Parent age	Type of active play
Parent sex	Independence of active play
Parent race/ethnicity	***Weather***
Parent nativity	Total rainfall
Parent education	Mean temperature
Household income	

#### Home and neighborhood characteristics

Questions in the BEAP Study questionnaire on each child’s home and neighborhood will collect information about the characteristics of the home and neighborhood, including (1) home building type; (2) presence of home yard; (3) sidewalk availability; and (4) street type. Data have shown that the availability of green space in the form of front or back yards or the presence of cul-de-sacs and sidewalks can be active play promoting environments [17,48-50].

#### Neighborhood accessibility

Participant walking access to recreational facilities, friend’s home and other locations or destinations for active play will be estimated using BEAP Study questionnaire responses. The number of minutes to walk to these locations from the home will be estimated to provide a characterization of the urban infrastructure distribution. Five types of destinations will be captured: (1) education; (2) transport; (3) recreation; (4) food retail; and (5) social.

### Outcome study variable

#### Child active play

Active play will be estimated by gathering information on frequency per week or month of active play inside the home, neighborhood and other locations (e.g., public playground). A single outcome variable of active play will be constructed using participant responses to two questionnaire items: 1) “For the past seven days, how many minutes per day has your child participated in active play?” and 2) “Over a typical or usual week, how many days has your child participated in active play for a total of at least 60 minutes per day?”.

### Study covariates

#### Demographics

Parent and child age, sex, race/ethnicity, nativity and highest level of education will be collected from the participants through the BEAP Study questionnaire. Data on the total annual household income will also collected.

#### Body size and health conditions

In the absence of anthropometric measurement collection for body mass indices (BMI), self-reported height and weight measurements are often collected from participants. As an alternative to anthropometrics, there are limitations with these self-reported measurements, such as bias and poor accuracy, which may be a result of social desirability or difficulties with recall [51]. However, if self-reported measurements are corrected for biases associated with sociodemographic characteristics of the study respondents, these data can prove to be very useful [52]. Parent and child BMIs will be estimated using height and weight BEAP Study questionnaire responses. BMI will be calculated by dividing weight in pounds by height in inches squared and multiplying by a conversion factor of 703. For adults 20 years old and older, BMI will be interpreted using standard weight status categories that are the same for all ages and both sexes [53]. For children, the interpretation of BMI will use both age- and sex-specific categories [54]. Data on pre-existing medical conditions that may affect a child’s ability to engage in active play or that may be related to physical inactivity will also be collected.

#### Children’s availability and use of electronic devices

The availability of electronics (e.g., television) in the child’s bedroom and the ownership of personal electronics (e.g. iPad) will qualitatively determine the level of electronic usage. Additionally, questions in the BEAP Study questionnaire will subjectively assess the number of minutes per day of various sedentary behaviors (e.g., watching television).

#### Child active commuting

For an average week, the frequency and duration of active commuting trips to or from school for the child will be estimated using responses from the BEAP Study questionnaire. The BEAP Study will assess three modes of active commute: (1) walk; (2) bike; and (3) public transportation.

#### Dog ownership

Research has demonstrated that dog ownership is associated with children’s walking and physical activity. A recent study found that dog ownership was associated with, on average, 29 more minutes of walking and 142 more minutes of physical activity per week among children [55]. For this study, the number of days per week for dog walking and playing with the dog will be quantified based on BEAP Study questionnaire data.

#### Parent rules

Since the goal of the BEAP Study is to model neighborhood playability by characterizing and estimating children’s active play, capturing an understanding of parental rules, which may act as an active play barrier, is essential. Questions on the enforcement of homework (e.g., Do homework before going outside), electronics use (e.g., Hours per day of television) and outdoor play (e.g., Stay in neighborhood) rules will collect these data in the BEAP Study questionnaire.

#### Parent neighborhood perceptions

Parent neighborhood perceptions can play a significant role in the level of outdoor physical activity among children [17]. Research has shown that children whose parents perceived their neighborhoods as unsafe watched more television and participated in less physical activity [56]. The relationship between parent neighborhood perception and childhood physical activity has varied based on age, sex, two-parent household and urbanicity [57-64]. Through a series of statements in the BEAP Study questionnaire, parent neighborhood perception of safety and the neighborhood quality of physical activity promotion will be assessed.

#### Parent and peer physical activity

Early research has demonstrated a positive relationship between parent and child physical activity. For example, when both parents are active, the children can be 5.8 times as likely to be active [65]. Other studies have found a positive relationship with parent supportive behaviors for their child’s physical activity and child physical activity [66]. Although the positive relationship between parent and child physical activity has not been consistently demonstrated, the BEAP Study will explore this relationship [67-70]. Data will be collected on parent occupational and recreational physical activity in the BEAP Study questionnaire.

#### Active play qualifiers

Active play will be characterized by gathering information on location (e.g. home) and type (e.g., playing catch) of active play. Data on the participation of active play with peers, sibling or parents, as well as, the participation of school or community sport teams will be collected to determine the level of independent active play.

#### Weather

The impact of weather conditions on physical activity in children has been explored. Although conclusions regarding the effect modification of weather have been inconclusive, these variables should not be overlooked [71,72]. By using the return date of the questionnaire as a set point, data on the total rainfall and mean temperature at the nearest land-based observation station during the preceding seven days will be obtained from the National Oceanic and Atmospheric Administration (NOAA) Climatic Data Center in order to monitor the potential confounding effect of weather conditions. The occurrence of active play on warmer days or weekends will also be assessed.

### Statistical analysis and bias

The primary goal of the BEAP Study is to develop a playability index or score by establishing the relationship between active play in children and various measurements of their neighborhood built environment after adjustment for potential confounding. To this end, an ordered logit model will be used regressing an ordinal active play outcome on built environment exposure variables. Associations between exposure (built environment) and outcome (active play) variables will be estimated based on data derived from the participant BEAP Study questionnaire and objective built environment measures, such as land use data and GIS, in order to model neighborhood playability scores. Any incomplete BEAP Study questionnaire data will be excluded from analysis and assumed to be randomly missing.

Demographic variables will automatically be added as covariates in this primary model. Due to the large number of other potential confounders measured in the BEAP Study, a systematic approach will be used to select additional variables for inclusion in the model. First, a correlation matrix will be built and used to select covariates that show an association with active play. Akaike Information Criterion (AIC), a penalized measure of model fit, will be used to determine which of these covariates will be included in the final model based on the trade-off between the model’s goodness of fit and complexity. Following the construction of the final model, the predictor coefficients will be used as parameters in the playability scoring system while weighing the various built environment predictors relative to their adjusted association with childhood active play.

### Ethics and dissemination

The BEAP Study received ethical approval from the Uniformed Services University of Health Sciences, Human Research Protections Program Office (USUHS IRB). The face page of the BEAP Study questionnaire will contain the information generally found in a consent form. There will be a statement on that face page that will indicate that by answering the questions and returning the questionnaire in the postage-paid self-addressed envelope, the participant is providing and documenting his/her consent. The Principal Investigator (PI) will annotate the questionnaire to the effect informed consent was received. The mailed BEAP Study questionnaire will also encourage the alternative option of completing the identical online version of the questionnaire via Qualtrics.com with the provided secure and encrypted web address and unique access code. With the online questionnaire version, the same consent information will be provided with the addition of a statement indicating that the participant’s IP address will remain unknown. Similar to the mail version, the online questionnaire version will provide a statement indicating that by beginning the questionnaire, the participant acknowledges that he/she has read this information and agreed to participate in this research with the knowledge that he/she is free to withdraw participation any time without penalty. The consent information is written at a reading level that is easily understood by all.

All data will be stored in a secure and password protected location prior to and after data entry. The data entered through the online questionnaire via Qualtrics.com will use Transport Layer (TLS) encryption (also known as HTTPS) for all transmitted data. The data will be secured and encrypted in the user account. Qualtrics will not have ownership of the data and Qualtrics employees will not have access to the data. Only the designated researchers listed in the USUHS IRB will have access to the data. Participation in this study will be anonymous and each questionnaire will have a unique identification number. Data collected from the study will be stored for up to 5 years and then all identifying information will be destroyed and discarded.

At the conclusion of this study, written reports of the key findings will be provided to all study participants. BEAP results will also be disseminated through peer-review publications and conference presentations. Key stakeholders, government and non-government organizations will also have access to study findings and recommendations resulting from this research through newsletters, seminars and an official BEAP Study website.

## Discussion

We have described the BEAP Study methodology which intends to generate a neighborhood playability index by characterizing and quantifying children’s active play using parent-reported physical activity data in children, GIS data and built environment objective measures in participant neighborhoods. A unique facet of this study is the examination of the relationship between parent-reported physical activity, parent perception, and built environment objective measures specific to DMV neighborhoods. Compared to other areas, such as Seattle, Washington and San Diego, California, where similar research has been performed, the DMV holds over 25% Black residents [73]. In contrast, the proportion of Black residents in Seattle and San Diego is 5% and 6%, respectively [74,75]. This is a unique aspect of the DMV demography particularly since over 35% of Black girls and boys are obese or overweight [1]. While San Diego has a large Hispanic population of 30%, another ethnic group with a childhood obesity and overweight prevalence over 35%, the DMV area maintains a healthy population size of both racial and ethnic groups [1,75]. Interestingly, the minimum level of educational attainment, which may influence household physical activity levels and parental neighborhood perceptions, in the DMV area is higher than the country’s average. With respect to post-graduate degree attainment, the rate is 23%, compared to 14%, 17% and 11% in Seattle, San Diego and the entire United States, respectively. In addition to the unique socioeconomic diversity, the DMV neighborhoods represent an area comprised of varying degrees of sprawl, urbanization, single-use and low-density zoning, transit developments, housing subdivisions, protected green spaces and other elements that create the building blocks of a built environment. As outlined, the associations between these variables will be achieved through several measures, such as the BEAP Study questionnaire and publically available data, to promote awareness in this critical area of obesity related research, policy advancement, and most importantly health outcomes of children and adolescents.

In recent research, long-term physical and mental health outcomes have been demonstrated to improve in children who participate in social interactions that involve physical activities [11]. Associations have also been identified between residential placement in poor quality built environment and adverse health outcomes including reduced levels physical activity [12-17]. Previous research has demonstrated that proximity to recreational facilities, parks, and schools was positively association with physical activity levels in adolescents [76], whereas negative local conditions (e.g., traffic hazards, crime, area deprivation) had a negative association [77,78]. Exploring and understanding these associations between the built environment and active play or playability has become even more important because of the significant increase in childhood obesity throughout the U.S. This study will further support the need to continue research in this area in order to fully understand the complexities surrounding childhood overweight and obesity.

The BEAP Study will improve our understanding of the built environment and childhood playability relationship while also adding a new and unexplored knowledge base of this research within the DMV area. Subjective variables, such as parental neighborhood perception, may also have a significant influence on childhood active play and thus neighborhood playability scores. A unique feature of the BEAP Study is that it will take place in the DMV and a study of this type has not been conducted within the DMV area, one that maintains a mosaic of built environments leveraged on race/ethnicity, income, education, nativity and many other socioeconomic factors. Hence, these factors and others provide the foundation for subjectivities, which can dictate parental choices, such as where one lives or if and where children are permitted to play. This research will provide additional insight to the bi-directional connection and dependence of subjective parental choices, objective built environment measures and childhood active play.

The BEAP Study will contribute to the body of evidence-based built environment and physical activity research by creating a DMV research arm. As this study should result in an abundance of rich data that will be of interest to secondary or auxiliary aims, there exists the possibility of additional analyses. One such example is the relationship between objective measurements to subjective parental opinion of the built environment. Results from the BEAP Study and future research conducted in the DMV area may be generalized to both the national and international scale, as a variety of built environments (e.g. urban, suburban, rural) exist within this area.

The primary weakness of this study is the use of subjective, parent-reported measurements of physical activity. Thus, a future goal of this research is to continue exploring the aforementioned associations, however, with the use of objective physical activity measurements. Potential additional weaknesses include biased sampling of the selection of child households from a consumer marketing company and error in measuring neighborhood recreation space due to the technical difficulty in fully capturing all potential playable areas. One source of this difficulty may arise from the lack of quality GIS parks data, however, by appropriately adjusting for this and other study limitations, findings from the BEAP Study will not be significantly compromised.

Finally, by illustrating the association between the built environment and active play or playability and ultimately child health outcomes, local, regional, and national policy and land use planners may find utility to the research findings and conclusions of the BEAP Study. Engagement of policy and land use planners will ensure future improvements to neighborhood design and playability. Promulgation of the BEAP Study findings to all interested and influential parties will augment the potential impact of these findings on a national scale.

## Additional file

Additional file 1:
**BEAP study questionnaire.** PDF (Adobe Acrobat). BEAP study questionnaire.

## Abbreviations

(BEAP): Built environment and active play
(DMV): Washington, DC area (Including Maryland and Virginia)
(GIS): Geographic information system
(MVPA): Moderate-to-vigorous physical activity
(STROBE): Strengthening the reporting of observational studies in epidemiology
(NOAA): National oceanic and atmospheric administration
(AIC): Akaike information criterion
(HTTPS): Transport layer (TLS) encryption

## References

[1] Ogden CL, Carroll MD, Kit BK, Flegal KM (2014). Prevalence of childhood and adult obesity in the United States, 2011-2012. JAMA.

[2] NCHS (2012). With Special Features on Socioeconomic Status and Health:2011. U.S. Department of Health and Human Services.

[3] NSCH (2013) 2011/2012 - National Survey of Children’s Health - Virginia Child Health Measures. Available: http://www.childhealthdata.org. [Accessed 2014 August 20].

[4] Hedley AA, Ogden CL, Johnson CL, Carroll MD, Curtin LR, Flegal KM (2004). Prevalence of overweight and obesity among US children, adolescents, and adults, 1999-2002. JAMA.

[5] Skinner AC, Skelton JA (2014). Prevalence and trends in obesity and severe obesity among children in the United States, 1999-2012. JAMA Pediatr.

[6] Healthy People (2013). Physical Activity. HealthyPeople.gov.

[7] Troiano RP, Berrigan D, Dodd KW, Masse LC, Tilert T, McDowell M (2008). Physical activity in the United States measured by accelerometer. Med Sci Sports Exerc.

[8] Centers for Disease Control and Prevention CDC (2014). Youth Risk Behavior Surveillance — United States, 2013. MMWR Surveill Summ.

[9] DHHS (2008). 2008 physical activity guidelines for americans.

[10] NASPH (1999). The fitness equation: physical activity + balanced diet = Fit kids.

[11] Kelty SF, Zubrick SR, Giles-Corti B (2008). Healthy body, healthy mind: why physically active children are healthier physically, psychologically and socially.

[12] Galea S, Ahern J, Rudenstine S, Wallace Z, Vlahov D (2005). Urban built environment and depression: a multilevel analysis. J Epidemiol Community Health.

[13] Mair C, Diez Roux AV, Galea S (2008). Are neighbourhood characteristics associated with depressive symptoms? a review of evidence. J Epidemiol Community Health.

[14] Weich S, Blanchard M, Prince M, Burton E, Erens B, Sproston K (2002). Mental health and the built environment: cross-sectional survey of individual and contextual risk factors for depression. Br J Psychiatry.

[15] Frank LD, Saelens BE, Chapman J, Sallis JF, Kerr J, Glanz K (2012). Objective assessment of obesogenic environments in youth: geographic information system methods and spatial findings from the neighborhood impact on kids study. Am J Prev Med.

[16] Saelens BE, Sallis JF, Frank LD, Couch SC, Zhou C, Colburn T (2012). Obesogenic neighborhood environments, child and parent obesity: the neighborhood impact on kids study. Am J Prev Med.

[17] Tappe KA, Glanz K, Sallis JF, Zhou C, Saelens BE (2013). Children’s physical activity and Parents’ perception of the neighborhood environment: neighborhood impact on kids study. Int J Behav Nutr Phys Act.

[18] Laxer RE, Janssen I (2013). The proportion of youths’ physical inactivity attributable to neighbourhood built environment features. Int J Health Geogr.

[19] Van Dyck D, Cardon G, Deforche B, De Bourdeaudhuij I (2009). Lower neighbourhood walkability and longer distance to school are related to physical activity in Belgian adolescents. Prev Med.

[20] Mecredy G, Pickett W, Janssen I (2011). Street connectivity is negatively associated with physical activity in Canadian youth. Int J Environ Res Public Health.

[21] Sallis JF, Saelens BE, Frank LD, Conway TL, Slymen DJ, Cain KL (2009). Neighborhood built environment and income: examining multiple health outcomes. Soc Sci Med.

[22] Owen N, Cerin E, Leslie E, duToit L, Coffee N, Frank LD (2007). Neighborhood walkability and the walking behavior of Australian adults. Am J Prev Med.

[23] Van Dyck D, Cardon G, Deforche B, Sallis JF, Owen N, De Bourdeaudhuij I (2010). Neighborhood SES and walkability are related to physical activity behavior in Belgian adults. Prev Med.

[24] Sundquist K, Eriksson U, Kawakami N, Skog L, Ohlsson H, Arvidsson D (2011). Neighborhood walkability, physical activity, and walking behavior: the Swedish Neighborhood and Physical Activity (SNAP) study. Soc Sci Med.

[25] Lopez RP, Hynes HP (2006). Obesity, physical activity, and the urban environment: public health research needs. Environ Health.

[26] Gilmour H (2007). Physically active Canadians. Health Rep.

[27] Telama R, Yang X, Viikari J, Valimaki I, Wanne O, Raitakari O (2005). Physical activity from childhood to adulthood: a 21-year tracking study. Am J Prev Med.

[28] USDA. Encourage Active Play and Participate with Children. U.S. Department of Agriculture, U.S. Department of Health and Human Services. 2013. Available: http://www.fns.usda.gov/sites/default/files/participate.pdf. [Accessed 2015 February 2].

[29] Kilpatrick M, Hebert E, Bartholomew J (2005). College students’ motivation for physical activity: differentiating men’s and women’s motives for sport participation and exercise. J Am Coll Health.

[30] Puhl RM, Heuer CA (2010). Obesity stigma: important considerations for public health. Am J Public Health.

[31] Dumith SC, Gigante DP, Domingues MR, Kohl HW (2011). Physical activity change during adolescence: a systematic review and a pooled analysis. Int J Epidemiol.

[32] Matthews CE, Chen KY, Freedson PS, Buchowski MS, Beech BM, Pate RR (2008). Amount of time spent in sedentary behaviors in the United States, 2003-2004. Am J Epidemiol.

[33] Mitchell JA, Pate RR, Beets MW, Nader PR (2013). Time spent in sedentary behavior and changes in childhood BMI: a longitudinal study from ages 9 to 15 years. Int J Obes (Lond).

[34] Kneeshaw-Price S, Saelens BE, Sallis JF, Glanz K, Frank LD, Kerr J (2013). Children’s objective physical activity by location: why the neighborhood matters. Pediatr Exerc Sci.

[35] Neighborhood Impact on Kids (NIK) Project. 2013. Available: http://www.nikproject.org/About_Us.htm. [Accessed 2014 February 11].

[36] Neighborhood Impact on Kids (NIK) Surveys. 2013. Available: http://www.seattlechildrens.org/research/child-health-behavior-and-development/saelens-lab/measures-and-protocols/. [Accessed 2014 February 11].

[37] StreetSmartWalkScore (2007). Street Smart Walk Score: Methodology.

[38] van Loon J, Frank LD, Nettlefold L, Naylor PJ (2014). Youth physical activity and the neighbourhood environment: examining correlates and the role of neighbourhood definition. Soc Sci Med.

[39] Census (2013). American fact finder.

[40] Dillman DA, Phelps G, Tortora R, Swift K, Kohrell J, Berck J (2009). Response rate and measurement differences in mixed-mode surveys using mail, telephone, interactive voice response (IVR) and the internet. Soc Sci Res.

[41] Moudon AV, Lee C, Cheadle AD, Johnson D, Schmid TL, Weathers RD (2006). Operational definitions of walkable neighborhood: theoretical and empirical insights. J Phys Activity Health.

[42] Randall TA, Baetz BW (2001). Evaluating pedestrian connectivity for suburban sustainability. J Urban Plann Dev.

[43] Moudon AV, Hess P, Snyder MC, Stanilov K (1997). Effects of site design on pedestrian travel in mixed-use, medium-density environments. Transp Res Record.

[44] Jones E (2001). Liveable neighborhoods. World Transp Policy Pract.

[45] Berrigan D, Pickle LW, Dill J (2010). Associations between street connectivity and active transportation. Int J Health Geogr.

[46] Ewing R, Cervero R (2010). Travel and the built environment. J Am Plan Assoc.

[47] Forsyth A, Oakes JM, Schmitz KH, Hearst M (2007). Does residential density increase walking and other physical activity?. Urban Stud.

[48] Durand CP, Dunton GF, Spruijt-Metz D, Pentz MA (2012). Does community type moderate the relationship between parent perceptions of the neighborhood and physical activity in children?. Am J Health Promot.

[49] Mecredy G, Janssen I, Pickett W (2012). Neighbourhood street connectivity and injury in youth: a national study of built environments in Canada. Inj Prev.

[50] Spurrier NJ, Magarey AA, Golley R, Curnow F, Sawyer MG (2008). Relationships between the home environment and physical activity and dietary patterns of preschool children: a cross-sectional study. Int J Behav Nutr Phys Act.

[51] Yoong SL, Carey ML, D’Este C, Sanson-Fisher RW (2013). Agreement between self-reported and measured weight and height collected in general practice patients: a prospective study. BMC Med Res Methodol.

[52] Stommel M, Schoenborn CA (2009). Accuracy and usefulness of BMI measures based on self-reported weight and height: findings from the NHANES & NHIS 2001-2006. BMC Public Health.

[53] Centers for Disease Control and Prevention (CDC). About BMI for Adults. 2011. Available: http://www.cdc.gov/healthyweight/assessing/bmi/adult_bmi/index.html - Interpreted. [Accessed 2014 May 27].

[54] Centers for Disease Control and Prevention (CDC) (2011). About BMI for Children and Teens.

[55] Christian H, Trapp G, Lauritsen C, Wright K, Giles-Corti B (2013). Understanding the relationship between dog ownership and children’s physical activity and sedentary behaviour. Pediatr Obes.

[56] Datar A, Nicosia N, Shier V (2013). Parent perceptions of neighborhood safety and children’s physical activity, sedentary behavior, and obesity: evidence from a national longitudinal study. Am J Epidemiol.

[57] Weir LA, Etelson D, Brand DA (2006). Parents’ perceptions of neighborhood safety and children’s physical activity. Prev Med.

[58] Gorely T, Atkin AJ, Biddle SJ, Marshall SJ (2009). Family circumstance, sedentary behaviour and physical activity in adolescents living in England: Project STIL. Int J Behav Nutr Phys Act.

[59] Hohepa M, Scragg R, Schofield G, Kolt GS, Schaaf D (2007). Social support for youth physical activity: Importance of siblings, parents, friends and school support across a segmented school day. Int J Behav Nutr Phys Act.

[60] Sweeney NM, Glaser D, Tedeschi C (2007). The eating and physical activity habits of inner-city adolescents. J Pediatr Health Care.

[61] Hesketh K, Crawford D, Salmon J (2006). Children’s television viewing and objectively measured physical activity: associations with family circumstance. Int J Behav Nutr Phys Act.

[62] Bagley S, Salmon J, Crawford D (2006). Family structure and children’s television viewing and physical activity. Med Sci Sports Exerc.

[63] Lindquist CH, Reynolds KD, Goran MI (1999). Sociocultural determinants of physical activity among children. Prev Med.

[64] Moore JB, Beets MW, Kaczynski AT, Besenyi GM, Morris SF, Kolbe MB (2013). Sex moderates associations between perceptions of the physical and social environments and physical activity in youth. Am J Health Promot.

[65] Moore LL, Lombardi DA, White MJ, Campbell JL, Oliveria SA, Ellison RC (1991). Influence of parents’ physical activity levels on activity levels of young children. J Pediatr.

[66] Trost SG, Sallis JF, Pate RR, Freedson PS, Taylor WC, Dowda M (2003). Evaluating a model of parental influence on youth physical activity. Am J Prev Med.

[67] Craig CL, Cameron C, Tudor-Locke C (2013). Relationship between parent and child pedometer-determined physical activity: a sub-study of the CANPLAY surveillance study. Int J Behav Nutr Phys Act.

[68] Sigmundova D, Sigmund E, Vokacova J, Kopkova J (2014). Parent-child associations in pedometer-determined physical activity and sedentary behaviour on weekdays and weekends in random samples of families in the czech republic. Int J Environ Res Public Health.

[69] Jago R, Sebire SJ, Wood L, Pool L, Zahra J, Thompson JL (2014). Associations between objectively assessed child and parental physical activity: a cross-sectional study of families with 5-6 year old children. BMC Public Health.

[70] Hesketh KR, Goodfellow L, Ekelund U, McMinn AM, Godfrey KM, Inskip HM (2014). Activity levels in mothers and their preschool children. Pediatrics.

[71] Chan CB, Ryan DA, Tudor-Locke C (2006). Relationship between objective measures of physical activity and weather: a longitudinal study. Int J Behav Nutr Phys Act.

[72] Tucker P, Gilliland J (2007). The effect of season and weather on physical activity: a systematic review. Public Health.

[73] Census Reporter (2013). Washington-Arlington-Alexandria.

[74] Census Reporter (2013). Seattle-Tacoma-Bellevue.

[75] CensusReporter (2013). San Diego, CA. U.S. Census Bureau.

[76] Rodriguez DA, Cho GH, Evenson KR, Conway TL, Cohen D, Ghosh-Dastidar B (2012). Out and about: association of the built environment with physical activity behaviors of adolescent females. Health Place.

[77] Lowry R, Lee SM, Fulton JE, Demissie Z, Kann L (2013). Obesity and other correlates of physical activity and sedentary behaviors among US high school students. J Obes.

[78] Davison KK, Lawson CT (2006). Do attributes in the physical environment influence children’s physical activity? A review of the literature. Int J Behav Nut Phys Act.

