# Supplementary material for: Built environment and active play among Washington DC metropolitan children: A protocol for a cross-sectional study
Source: Arch Public Health. 2015 Apr 27;73(1):22. doi: 10.1186/s13690-015-0070-3 (PMC4436840; doi:10.1186/s13690-015-0070-3)
Supplement: Additional file 1: — BEAP study questionnaire. PDF (Adobe Acrobat). BEAP study questionnaire. [file 13690_2015_70_MOESM1_ESM.pdf]

UNIFORMED SERVICES UNIVERSITY
of the Health Sciences

Neighborhood Play Questionnaire

Thank you for considering our request to complete and return this anonymous questionnaire. Participation in this research is voluntary, but we hope you will participate because we need to hear from a wide range of people to improve our understanding of this topic. We are asking participants to try to complete and return their questionnaires within 7 days. We do not have your name and will only ask for your name at the end of this questionnaire if you are interested in participating in a follow-up research study. At the conclusion of this research, your address and all other identifying information will be destroyed and discarded. A summary report of what we have learned from all the people who return their questionnaires will be shared with scientists and policy makers who work on neighborhood and physical activity issues.

As a token of appreciation for your participation, we have enclosed a $10.00 gift card. Through your participation, you will be contributing to the knowledge about the relationship between neighborhoods and childhood physical activity. No risks or discomforts are anticipated from taking part in this research study.

We encourage you to use the identical online version to complete this questionnaire at the following secure and encrypted web address [redacted] using the access code printed in the top right corner of this page. However, if you prefer, please complete and return this provided paper version. Only one version is needed. We estimate that it will take you about 15-20 minutes to complete this questionnaire. If you complete this paper version questionnaire, simply return it in the stamped, self-addressed envelope we have provided. By completing and returning this questionnaire, you are providing and documenting your consent. If you have any questions, please contact Dr. Jennifer D. Roberts, who is the researcher in charge of this research study at [redacted].

Thank you for helping us with this important project!

What to do: Please read each question and put a mark (**X**) beside your answer. Only mark one response for each question, unless you are asked to "check all that apply".

- All information is strictly confidential
- Please respond only about 1 child who is 7 to 12 years of age and living in your home
- Provide only one answer for each question unless otherwise noted
- If at any point you feel uncomfortable, you can skip a question or stop completely

Questions About Your Family

1. Can you please confirm your home address?

**Street Address**	
**City**	
**State**	
**Zip Code**	

2. Do you have at least one child living in your home that is 7 to 12 years of age?

☐ YES: PLEASE COMPLETE THE SURVEY.

☐ NO: YOU HAVE COMPLETED THE SURVEY. THANK YOU!

3. Has the above address been your child's residence for at least 5 days per week for most of the past year?

☐ YES

☐ NO

4. How many children are living in your home that are 7 to 12 years of age?

☐ 1

☐ MORE THAN 1

For the remainder of this questionnaire, please respond only about the child in your home who is **7 to 12 years of age** and who had **the most recent birthday**.

Think about your **child's activities** over the **PAST YEAR** when answering questions, unless otherwise specified. Please mark one response for each item.

Questions About Child's Bedroom, Personal Electronics and Play Equipment

5. **Please indicate whether the following are currently or have been <u>in your child's bedroom or the room in which he or she sleeps</u>.**

	YES	NO
a. TV	☐	☐
b. VCR or DVD player	☐	☐
c. Computer	☐	☐
d. Video game system (non-hand held—Playstation, Xbox, etc)	☐	☐

6. **Does your child have the following for his/her own use?**

	YES	NO
a. Cell phone or 2-way radio	☐	☐
b. Hand held videogame players (Game Boy, Sony PSP, etc)	☐	☐
c. Tablet (iPad, Kindle, etc.)	☐	☐
d. Portable music players (radio, MP3 or iPod)		

7. **How often does your child use these items at or around the home (or in a common apartment area)?**

	NOT AVAILABLE (DON'T HAVE)	AVAILABLE BUT NEVER USE	USE ONCE A MONTH OR LESS	USE ONCE EVERY OTHER WEEK	USE ONCE A WEEK OR MORE
a. Bike	☐	☐	☐	☐	☐
b. Basketball hoop	☐	☐	☐	☐	☐
c. Jump rope	☐	☐	☐	☐	☐
d. Active video games (e.g. with dance pad, Wii, etc.)	☐	☐	☐	☐	☐
e. Sports equipment (like balls, racquets, bats, sticks)	☐	☐	☐	☐	☐
f. Swimming pool	☐	☐	☐	☐	☐
g. Roller skates, skateboard, scooter	☐	☐	☐	☐	☐
h. Fixed play equipment (e.g., swing set, play house, jungle gym)	☐	☐	☐	☐	☐

Questions About Your Child's Active Play

"Active Play" is defined as participating in <u>vigorous-intensity</u> or <u>moderate-intensity</u> physical activities for fun and enjoyment in an official (For example: team sports) or unofficial capacity (For example: neighborhood game of basketball).

<u>Vigorous-intensity activities</u> require hard physical effort. These activities make your child's heart beat A LOT faster than normal and cause him or her to breathe A LOT harder than normal.

<u>Moderate-intensity activities</u> require moderate physical effort. These activities make your child's heart beat A LITTLE faster than normal and cause him or her to breathe A LITTLE harder than normal.

<u>Examples of Vigorous-Intensity or Moderate-Intensity Activities</u>

Include, but are not limited to, games involving running and chasing, such as tag; jumping rope; running; brisk walking; throwing and catching with a partner; hiking; skateboarding; in-line skating or sports, such as baseball or softball, ice, street or field hockey, basketball, swimming or tennis; bicycle riding; gymnastics; rope or tree climbing; swinging, hanging or climbing on playground equipment or games, such as tug-of-war

8. To what extent do you agree or disagree with the following statement:

My child has a disability or condition that may limit his or her ability to participate in active play?

- ☐ STRONGLY AGREE
- ☐ AGREE
- ☐ DISAGREE
- ☐ STRONGLY DISAGREE
- ☐ DON'T KNOW/NOT SURE

PHYSICAL ACTIVITY AT HOME AND IN THE NEIGHBORHOOD

9. Throughout the year, how often does your child participate in active play inside your home?

	NEVER	ONCE A MONTH OR LESS	ONCE EVERY OTHER WEEK	ONCE A WEEK	2 OR 3 TIMES A WEEK	4 OR MORE TIMES A WEEK
a. Inside your home	☐	☐	☐	☐	☐	☐

10. How often does your child participate in active play in/at the following places throughout the year?

	NEVER	ONCE A MONTH OR LESS	ONCE EVERY OTHER WEEK	ONCE A WEEK	2 OR 3 TIMES A WEEK	4 OR MORE TIMES A WEEK
a. Your yard or common area	☐	☐	☐	☐	☐	☐
b. Your driveway or alley	☐	☐	☐	☐	☐	☐
d. At a neighbor's house or yard	☐	☐	☐	☐	☐	☐

e. A local street, sidewalk, or vacant lot	☐	☐	☐	☐	☐	☐
f. Nearby cul-de-sac or dead-end street	☐	☐	☐	☐	☐	☐
g. Other neighborhood area	☐	☐	☐	☐	☐	☐

11. For the past seven days, how many minutes per day has your child participated in active play in/at any of the places listed in question #10?

- ☐ 0 MINUTES
- ☐ 1-15 MINUTES
- ☐ 16-30 MINUTES
- ☐ 31-45 MINUTES
- ☐ 46-60 MINUTES
- ☐ MORE THAN 60 MINUTES

12. Over a typical or usual week, how many days has your child participated in active play for a total of at least 60 minutes per day in/at any of the places listed in question #10?

NONE	1 DAY	2 DAYS	3 DAYS	4 DAYS	5 DAYS	6 DAYS	7 DAYS
☐	☐	☐	☐	☐	☐	☐	☐

13. Have you ever noticed your child being out of breath (For example: breathing a little or a lot harder than normal) during or after his or her participation in active play in/at any of the places listed in question #10?

- ☐ YES
- ☐ NO
- ☐ DON'T KNOW/NOT SURE

14. Does your child participate in any of the following active play activities in/at any of the places listed in question #10?

	YES	NO
a. Running or chasing	☐	☐
b. Climbing	☐	☐
c. Playing catch	☐	☐
d. Skateboarding	☐	☐
e. Soccer	☐	☐
f. Basketball	☐	☐
g. Baseball/Softball		
h. Other ____________________(PLEASE SPECIFY)	☐	☐

PHYSICAL ACTIVITY AT OTHER LOCATIONS

15. Throughout the year how often does your child participate in active play in/at the following locations?

	NEVER	ONCE A MONTH OR LESS	ONCE EVERY OTHER WEEK	ONCE A WEEK	2 OR 3 TIMES A WEEK	4 OR MORE TIMES A WEEK
a. Indoor recreation or exercise facility (e.g. YMCA or gym)	☐	☐	☐	☐	☐	☐
b. Beach, lake, river or creek	☐	☐	☐	☐	☐	☐
c. Bike/hiking/walking trails and paths	☐	☐	☐	☐	☐	☐
d. Basketball court	☐	☐	☐	☐	☐	☐
e. Soccer field						
f. Other playing fields/court	☐	☐	☐	☐	☐	☐
g. Indoor swimming pool	☐	☐	☐	☐	☐	☐
h. Public park	☐	☐	☐	☐	☐	☐
i. Public open space that is not a park	☐	☐	☐	☐	☐	☐
j. Public playground	☐	☐	☐	☐	☐	☐
k. School grounds during non-school hours	☐	☐	☐	☐	☐	☐
l. Outdoor swimming pool	☐	☐	☐	☐	☐	☐
m. Ski or other winter area	☐	☐	☐	☐	☐	☐
n. Other ____________________ (PLEASE SPECIFY)	☐	☐	☐	☐	☐	☐

16. For the <u>past seven days</u>, how many <u>minutes per day</u> has your child participated in active play in/at any of the locations listed in question #15?

☐ 0 MINUTES
☐ 1-15 MINUTES
☐ 16-30 MINUTES
☐ 31-45 MINUTES
☐ 46-60 MINUTES
☐ MORE THAN 60 MINUTES

17. **Over a <u>typical or usual week</u>, how many days has your child participated in active play for a total of <u>at least 60 minutes per day</u> in/at any of the locations listed in question #15?**

NONE	1 DAY	2 DAYS	3 DAYS	4 DAYS	5 DAYS	6 DAYS	7 DAYS
☐	☐	☐	☐	☐	☐	☐	☐

18. **Have you ever noticed your child being out of breath (For example: breathing a little or a lot harder than normal) during or after his or her participation in active play in/at any of the locations listed in question #15?**

☐ YES
☐ NO
☐ DON'T KNOW/NOT SURE

19. **Does your child participate in active play activities more often during warmer days of the year?**

☐ YES
☐ NO
☐ DON'T KNOW/NOT SURE

20. **Does your child participate in active play activities more often on weekends compared to weekdays?**

☐ YES
☐ NO
☐ DON'T KNOW/NOT SURE

<u>ACCESS TO PHYSICAL ACTIVITY AND ACTIVITY WITH FRIENDS OR FAMILY</u>

21. **How many <u>days per week</u> does your child usually walk or bike to/from the following?**

	NEVER	ONCE A MONTH OR LESS	ONCE EVERY OTHER WEEK	ONCE A WEEK	2 OR 3 TIMES A WEEK	4 OR MORE TIMES A WEEK
a. Indoor recreation or exercise facility	☐	☐	☐	☐	☐	☐
b. Friend's or relative's house	☐	☐	☐	☐	☐	☐
c. Outdoor recreation place (e.g. park, playground)	☐	☐	☐	☐	☐	☐
d. Food store or restaurant	☐	☐	☐	☐	☐	☐
e. Other ____________________ (PLEASE SPECIFY)	☐	☐	☐	☐	☐	☐

22. **How do you rate your child's physical fitness compared to others of the same age and sex?**

MUCH LOWER	SOMEWHAT LOWER	ABOUT THE SAME LEVEL	SOMEWHAT HIGHER	MUCH HIGHER
☐	☐	☐	☐	☐

23. During <u>a typical week</u>, how often do your <u>child's siblings or friends</u>:

	NEVER	1-2 DAYS	3-4 DAYS	5-6 DAYS	EVERY DAY
a. Participate in active play with your child?	☐	☐	☐	☐	☐
b. Ask your child to walk or bike to school or to a friend's house	☐	☐	☐	☐	☐

24. During <u>a typical week</u>, how often have <u>you or another adult in the household</u>:

	NEVER	1-2 DAYS	3-4 DAYS	5-6 DAYS	EVERY DAY
a. Participated in active play with your child?	☐	☐	☐	☐	☐

25. Does your child participate in a school or community sport team?

☐ YES
☐ NO
☐ DON'T KNOW/NOT SURE

NON-PHYSICAL ACTIVITY

26. During <u>a typical week</u>, how many days does your child sit and watch TV, play videogames on the computer, or with other electronic devices:

	NEVER	1-2 DAYS	3-4 DAYS	5-6 DAYS	EVERY DAY
a. Alone	☐	☐	☐	☐	☐
b. With siblings	☐	☐	☐	☐	☐
c. With parent or guardian	☐	☐	☐	☐	☐
d. With friends	☐	☐	☐	☐	☐

27. Please indicate how much time on <u>a typical weekday</u> your child does the following activities, when he or she is mostly sitting, and not moving around. Please think about the time from when your child wakes up until he or she goes to bed. Please <u>do not</u> include time when your child is in school during regular hours.

	NONE	15 MIN PER DAY	30 MIN PER DAY	1 HOUR PER DAY	2 HOURS PER DAY	3 HOURS PER DAY	4 HOURS OR MORE PER DAY
a. Watching television/videos/DVDs	☐	☐	☐	☐	☐	☐	☐
b. Playing sedentary computer or video games (e.g. Xbox, cell phone)	☐	☐	☐	☐	☐	☐	☐
c. Using Internet, e-mailing, or other electronic media for fun or relaxation	☐	☐	☐	☐	☐	☐	☐

d. Doing homework (including reading, writing, or using the computer)	☐	☐	☐	☐	☐	☐	☐
e. Reading a book or magazine <u>not</u> for school (including comic books)	☐	☐	☐	☐	☐	☐	☐
f. Riding in a car	☐	☐	☐	☐	☐	☐	☐

Questions About Your Child's Home

When answering the following questions, please think about the home in which you and your child live. This home should match the address you provided in question #1.

28. What type of building is your home/your child's home?

☐ DETACHED SINGLE FAMILY HOUSE
☐ TOWNHOUSE
☐ CONDOMINIUM OR APARTMENT BUILDING
☐ OTHER ____ (PLEASE SPECIFY)

29. Does your home/your child's home have any of the following?

	YES	NO
a. Front yard	☐	☐
b. Back yard	☐	☐
c. Side yard	☐	☐
d. Driveway	☐	☐

30. Does the part of your/your child's street that you live on have sidewalks?

☐ YES
☐ NO

31. Do the streets connected to the street of your home/your child's home have sidewalks?

☐ YES
☐ NO
☐ DON'T KNOW/NOT SURE

32. Do you rent or own your/your child's home?

☐ RENT
☐ OWN
☐ OTHER ____ (PLEASE SPECIFY)

33. Is your street/your child's street in a cul-de-sac or dead-end?

☐ YES
☐ NO

34. Is there a dog at your home/your child's home?

☐ YES: PLEASE ANSWER THE NEXT QUESTION

☐ NO: PLEASE SKIP TO QUESTION # 37

35. How many <u>days per week</u> did your child spend <u>walking your dog</u> last week (including with a parent)?

NONE	1 DAY	2 DAYS	3 DAYS	4 DAYS	5 DAYS	6 DAYS	7 DAYS
☐	☐	☐	☐	☐	☐	☐	☐

36. How many <u>days per week</u> did your child spend <u>playing outside with your dog</u> last week (not including walking)?

NONE	1 DAY	2 DAYS	3 DAYS	4 DAYS	5 DAYS	6 DAYS	7 DAYS
☐	☐	☐	☐	☐	☐	☐	☐

37. About how long would it take <u>you to walk</u> from your/your child's home to each of the <u>nearest places</u> listed below? Please check off the time it would take you to walk to each place, even if you do not really ever go there.

	1-5 MIN	6-10 MIN	11-20 MIN	21-30 MIN	OVER 30 MIN	DON'T KNOW
a. Indoor recreation or exercise facility (e.g. YMCA)	☐	☐	☐	☐	☐	☐
b. Beach, lake river or creek	☐	☐	☐	☐	☐	☐
c. Bike/hiking/walking trails and paths	☐	☐	☐	☐	☐	☐
d. Basketball court	☐	☐	☐	☐	☐	☐
e. Other playing fields/court (e.g. tennis, softball)	☐	☐	☐	☐	☐	☐
f. Indoor swimming pool	☐	☐	☐	☐	☐	☐
g. Public park	☐	☐	☐	☐	☐	☐
h. Public open space that is not a park	☐	☐	☐	☐	☐	☐
i. Friend's or relative's house	☐	☐	☐	☐	☐	☐
j. Public playground	☐	☐	☐	☐	☐	☐
k. School grounds during non-school hours	☐	☐	☐	☐	☐	☐
l. Outdoor swimming pool	☐	☐	☐	☐	☐	☐
m. Ski or other winter recreation area	☐	☐	☐	☐	☐	☐
n. Convenience/corner store	☐	☐	☐	☐	☐	☐
o. Library	☐	☐	☐	☐	☐	☐
p. Fast food restaurant	☐	☐	☐	☐	☐	☐
q. Bus or Metro stop	☐	☐	☐	☐	☐	☐

38. **How many <u>minutes per day</u> is your child's commute <u>to and from school</u> on an average school day?**

☐ 0 MINUTES
☐ 1-15 MINUTES
☐ 16-30 MINUTES
☐ 31-45 MINUTES
☐ 46-60 MINUTES
☐ MORE THAN 60 MINUTES

39. **In an <u>average school week</u>, how many days does your child use each of the following ways to get to and from school?**

Days per week TO school	NONE	1 DAY	2 DAYS	3 DAYS	4 DAYS	5 DAYS
a. Walk	☐	☐	☐	☐	☐	☐
b. Bike	☐	☐	☐	☐	☐	☐
c. Car	☐	☐	☐	☐	☐	☐
d. Bus or Metro	☐	☐	☐	☐	☐	☐
Days per week FROM school	**NONE**	**1 DAY**	**2 DAYS**	**3 DAYS**	**4 DAYS**	**5 DAYS**
a. Walk	☐	☐	☐	☐	☐	☐
b. Bike	☐	☐	☐	☐	☐	☐
c. Car/bus	☐	☐	☐	☐	☐	☐
d. Bus or Metro	☐	☐	☐	☐	☐	☐

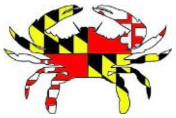

You're more than half-way complete!

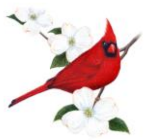

Questions About Parent/Guardian Rules

40. In your family, do you enforce any of the following rules about your child's homework and electronic use?

	YES	NO
a. Do homework before going outside	☐	☐
b. No television/DVD/computer before homework	☐	☐
c. Hours per day of television/DVD/computer<br>If yes, provide the maximum number of hours per weekday ________<br>If yes, provide the maximum number of hours per weekend day _____	☐	☐
d. Other<br>____(PLEASE SPECIFY)	☐	☐

41. Do you enforce any of the following rules about your child's activity?

	YES	NO
a. Stay within a certain distance of the house/parent	☐	☐
b. Do not go into the street	☐	☐
c. Come in before dark	☐	☐
d. Do not go places alone	☐	☐
e. Stay in the neighborhood	☐	☐
f. Do not ride bike in the street	☐	☐
g. Carry a cell phone or 2-way radio	☐	☐
h. Watch out for cars	☐	☐
i. Check in frequently by calling or coming home	☐	☐
j. Stay on paths, trails or sidewalk	☐	☐
k. Do not cross busy streets	☐	☐
l. Wear hat and/or sunscreen in summer	☐	☐
m. Do not fight with other kids	☐	☐
n. Do not vandalize other's or public property	☐	☐
o. Other<br>____(PLEASE SPECIFY)	☐	☐

Questions About Your Neighborhood

The following questions will ask about the home neighborhood in which you and your child live. When we say "local" and "within walking distance", we mean a 10-15 minute walk from your home.

42. Please mark the answer that best applies to you and your child's neighborhood.

	STRONGLY DISAGREE	SOMEWHAT DISAGREE	SOMEWHAT AGREE	STRONGLY AGREE
a. Many streets in my neighborhood are hilly	☐	☐	☐	☐
b. There are not any dead end streets	☐	☐	☐	☐
c. There are sidewalks on most streets	☐	☐	☐	☐
d. Usually sidewalks are separated from the road/traffic by parked cars	☐	☐	☐	☐
e. There are trees along the streets	☐	☐	☐	☐
f. There are many interesting things for my child to look at while walking	☐	☐	☐	☐
g. There are many natural things for my child to look at	☐	☐	☐	☐
h. There are many buildings/homes for my child to look at	☐	☐	☐	☐
i. The traffic makes it difficult or unsafe for my child to walk	☐	☐	☐	☐
j. The speed of traffic on most streets is usually 30 mph or less	☐	☐	☐	☐
k. Most motorist drive faster than the posted speed limits	☐	☐	☐	☐
l. I'm afraid of my child being taken or hurt by a stranger when he/she is outside without me	☐	☐	☐	☐
m. Streets have good lighting at night	☐	☐	☐	☐
n. Walkers and bikers can be easily seen by people in their homes	☐	☐	☐	☐
o. There are crosswalks and signals on busy streets	☐	☐	☐	☐
p. There is a high crime rate	☐	☐	☐	☐
q. It is likely that my child can be taken or hurt by a stranger in my neighborhood	☐	☐	☐	☐
r. It is likely that my child can be taken or hurt by a stranger in my yard, driveway, or common area	☐	☐	☐	☐
s. I'm afraid of my child being taken or hurt by a known "bad" person (adult or child) in my neighborhood	☐	☐	☐	☐
t. There is a lot of litter on the streets	☐	☐	☐	☐

u. There are many families that look like us in our neighborhood	☐	☐	☐	☐

43. Please mark the answer that best applies to the following statements:
My child can walk or bike to the closest local park or playground (alone or with someone) because:

	STRONGLY DISAGREE	SOMEWHAT DISAGREE	SOMEWHAT AGREE	STRONGLY AGREE
a. There are sidewalks or bike lanes	☐	☐	☐	☐
b. The route is simple	☐	☐	☐	☐
c. The route has good lighting when it’s dark outside	☐	☐	☐	☐
d. There are no dangerous crossings	☐	☐	☐	☐
e. My child does not get too hot and sweaty	☐	☐	☐	☐
f. Other children walk or bike	☐	☐	☐	☐
g. It is considered cool to walk or bike	☐	☐	☐	☐
h. My child does not have much stuff to carry	☐	☐	☐	☐
i. It is easier than me driving there on the way to something else	☐	☐	☐	☐
j. It involves very little planning ahead	☐	☐	☐	☐
k. There are areas to leave a bike safely	☐	☐	☐	☐
l. There are no stray dogs	☐	☐	☐	☐
m. It is not too far	☐	☐	☐	☐
n. My child would not have to walk/bike through high crime or unsafe areas	☐	☐	☐	☐

44. Please mark the answer that best applies to the following statements:
It is difficult for my child to be active in our home neighborhood because:

	STRONGLY DISAGREE	SOMEWHAT DISAGREE	SOMEWHAT AGREE	STRONGLY AGREE
a. There is no choice of activities	☐	☐	☐	☐
b. There is no play equipment (e.g. basketball hoop)	☐	☐	☐	☐
c. There is no adult supervision	☐	☐	☐	☐
d. There are no other children there	☐	☐	☐	☐
e. It is not safe because of crime	☐	☐	☐	☐
f. It is not safe because of traffic	☐	☐	☐	☐
g. It does not have good lighting	☐	☐	☐	☐

45. **Have you been the victim of a crime in your neighborhood?**

☐ YES
☐ NO

46. **Do you know someone who has been the victim of a crime in your neighborhood?**

☐ YES
☐ NO
☐ DON'T KNOW/NOT SURE

Questions About Your Physical Activity

The set of questions in this section asks about YOUR physical activity, not your child's.

In answering the following questions, vigorous-intensity activities are activities that require hard physical effort and cause LARGE increases in breathing or heart rate.

While moderate-intensity activities are activities that require moderate physical effort and causes SMALL increases in breathing or heart rate.

PHYSICAL ACTIVITY AT EMPLOYMENT

47. **As part of your paid work, do you do vigorous-intensity activities for at least 10 minutes continuously?**

☐ YES: PLEASE ANSWER THE NEXT QUESTION
☐ NO: PLEASE GO TO QUESTION #50

48. **In a typical week, how many days do you do vigorous-intensity activities for at least 10 minutes continuously, as part of your paid work?**

NONE	1 DAY	2 DAYS	3 DAYS	4 DAYS	5 DAYS	6 DAYS	7 DAYS
☐	☐	☐	☐	☐	☐	☐	☐

49. **On a typical day, how many minutes per day do you spend doing vigorous-intensity activities as part of your paid work?**

☐ 0 MINUTES
☐ 1-15 MINUTES
☐ 16-30 MINUTES
☐ 31-45 MINUTES
☐ 46-60 MINUTES
☐ MORE THAN 60 MINUTES

50. **As part of your paid work, do you do moderate-intensity activities for at least 10 minutes continuously?**

☐ YES: PLEASE ANSWER THE NEXT QUESTION
☐ NO: PLEASE GO TO QUESTION #53

51. **In a typical week, how many days do you do moderate-intensity activities for at least 10 minutes continuously, as part of your paid work?**

NONE	1 DAY	2 DAYS	3 DAYS	4 DAYS	5 DAYS	6 DAYS	7 DAYS
☐	☐	☐	☐	☐	☐	☐	☐

52. **On a typical day, how many minutes per day do you spend doing moderate-intensity activities as part of your paid work?**

☐ 0 MINUTES
☐ 1-15 MINUTES
☐ 16-30 MINUTES
☐ 31-45 MINUTES
☐ 46-60 MINUTES
☐ MORE THAN 60 MINUTES

RECREATIONAL PHYSICAL ACTIVITY

53. **Do you ever participate in vigorous-intensity sports, fitness, or leisure activities recreationally?**

☐ YES: PLEASE ANSWER THE NEXT QUESTION
☐ NO: PLEASE GO TO QUESTION #56

54. **In a typical week, how many days do you do vigorous-intensity sports, fitness, or leisure activities recreationally for at least 10 minutes continuously?**

NONE	1 DAY	2 DAYS	3 DAYS	4 DAYS	5 DAYS	6 DAYS	7 DAYS
☐	☐	☐	☐	☐	☐	☐	☐

55. **On a typical day, how many minutes per day do you spend doing vigorous-intensity sports, fitness, or leisure activities recreationally?**

☐ 0 MINUTES
☐ 1-15 MINUTES
☐ 16-30 MINUTES
☐ 31-45 MINUTES
☐ 46-60 MINUTES
☐ MORE THAN 60 MINUTES

56. **Do you ever participate in moderate-intensity sports, fitness, or leisure activities recreationally?**

☐ YES: PLEASE ANSWER THE NEXT QUESTION
☐ NO: PLEASE GO TO QUESTION #59

57. **In a typical week, how many days do you do moderate-intensity sports, fitness, or leisure activities recreationally for at least 10 minutes continuously?**

NONE	1 DAY	2 DAYS	3 DAYS	4 DAYS	5 DAYS	6 DAYS	7 DAYS
☐	☐	☐	☐	☐	☐	☐	☐

58. **On <u>a typical day</u>, how many <u>minutes per day</u> do you spend doing <u>moderate-intensity</u> sports, fitness, or leisure activities <u>recreationally</u>?**

- ☐ 0 MINUTES
- ☐ 1-15 MINUTES
- ☐ 16-30 MINUTES
- ☐ 31-45 MINUTES
- ☐ 46-60 MINUTES
- ☐ MORE THAN 60 MINUTES

General Questions About You And Your Child

For this next set of questions, please think about YOURSELF.

59. **How old are you?** ______________________ (YEARS)

60. **What is your gender?**

- ☐ MALE
- ☐ FEMALE

61. **Are you of Hispanic or Latino ethnicity?**

- ☐ YES
- ☐ NO
- ☐ DON'T KNOW/NOT SURE

62. **What is your race (select all that apply)?**

- ☐ AFRICAN AMERICAN OR BLACK
- ☐ AMERICAN INDIAN OR ALASKA NATIVE
- ☐ ASIAN AMERICAN
- ☐ NATIVE HAWAIIAN OR OTHER PACIFIC ISLANDER
- ☐ WHITE
- ☐ OTHER ____ (PLEASE SPECIFY)

63. **Were you born in the United States?**

- ☐ YES
- ☐ NO
- ☐ DON'T KNOW/NOT SURE

64. **What is your weight?** ____ (POUNDS)

65. **What is your height?** _________________ (FEET) _________________ (INCHES)

66. What was your highest education level completed?

☐ LESS THAN 8TH GRADE
☐ COMPLETED 8TH GRADE
☐ SOME HIGH SCHOOL
☐ COMPLETED HIGH SCHOOL OR GED EQUIVALENT
☐ SOME COLLEGE OR VOCATIONAL TRAINING
☐ COMPLETED BACHELOR'S DEGREE
☐ COMPLETED GRADUATE OR PROFESSIONAL DEGREE (E.G. MASTERS, PHD, MD)

67. How many hours per week do you work outside of the home?

☐ NONE
☐ LESS THAN PART TIME (1-15 HOURS)
☐ PART TIME (16-35 HOURS)
☐ FULL TIME (35+ HOURS)

68. What is your total annual household income? (Include income for all family members and all sources such as wages/salary, child support, alimony, etc.)

☐ LESS THAN $15,000
☐ $15,001 - $30,000
☐ $30,001 – $50,000
☐ $50,001 - $75,000
☐ $75,001 - $100,000
☐ $100,001 - $150,000
☐ $150,001 - $250,000
☐ $250,001 - $500,000
☐ MORE THAN $500,000
☐ DON'T KNOW/NOT SURE/REFUSED

69. Do you or someone in your household own a car?

☐ YES
☐ NO

70. Other than you, how many adults 18 years of age or older live in the household? ___________

For the next set of questions, please think about THE CHILD that you have been referring to when answering the first part of the questionnaire.

71. What is his or her birth month and year?

_____________________ (MONTH) _____________________ (YEAR)

72. How old is your child? ______________________ (YEARS)

73. What is your child's gender?

☐ MALE
☐ FEMALE

74. Is your child of Hispanic or Latino ethnicity?

☐ YES
☐ NO
☐ DON'T KNOW/NOT SURE

75. What is your child's race (select all that apply)?

☐ AFRICAN AMERICAN OR BLACK
☐ AMERICAN INDIAN OR ALASKA NATIVE
☐ ASIAN AMERICAN
☐ NATIVE HAWAIIAN OR OTHER PACIFIC ISLANDER
☐ WHITE
☐ OTHER ____ (PLEASE SPECIFY)

76. Was your child born in the United States?

☐ YES
☐ NO
☐ DON'T KNOW/NOT SURE

77. What is your child's weight? ____ (POUNDS)

78. What is your child's height? _________________ (FEET) _________________ (INCHES)

79. What is the highest school grade your child has completed?

☐ KINDERGARTEN
☐ 1ST GRADE
☐ 2ND GRADE
☐ 3RD GRADE
☐ 4TH GRADE
☐ 5TH GRADE
☐ 6TH GRADE
☐ MORE THAN 6TH GRADE
☐ NONE OF THE ABOVE

80. Has a doctor ever told you that your child has any of the following conditions?

	YES	NO
a. Anxiety	☐	☐
b. Asthma	☐	☐
c. ADHD or ADD	☐	☐
d. Depression	☐	☐
e. Diabetes	☐	☐
f. High blood pressure	☐	☐
g. High cholesterol or high triglycerides	☐	☐

h. Overweight or obese	☐	☐
p. Other ____(PLEASE SPECIFY)	☐	☐

Questions About Future Research

81. The researchers of this research study plan to conduct a follow-up research study. Would you be interested in participating in another research study questionnaire or interview?

☐ YES: PLEASE ANSWER THE FINAL QUESTION

☐ NO: YOU HAVE COMPLETED THE SURVEY. THANK YOU!

82. Please provide a way that the researchers could contact you for your participation in future research studies.

**Full Name**	
**Email Address**	
**Phone Number**	

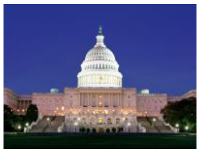

Thank you for filling out this questionnaire!

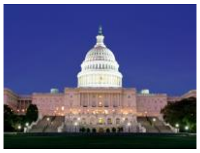

What to do next: If you have completed this questionnaire in its written format, please return your completed questionnaire in the provided stamped, self-addressed envelope. If you have lost or misplaced your provided envelope, please call Brandon Knight, the Research Coordinator at [redacted].
